# Thinking transdisciplinarily: Question-Collaboration Workshop to ideate across disciplines

**DOI:** 10.3389/fpsyg.2026.1773669

**Published:** 2026-05-05

**Authors:** Mayla R. Boguslav, Kayla de la Haye, Jennifer E. Cross

**Affiliations:** 1 Southern California Clinical and Translational Science Institute, University of Southern California, Los Angeles, CA, United States; 2 Department of Mathematics, College of Natural Sciences, Colorado State University, Fort Collins, CO, United States; 3 Center for Economic and Social Research, Dornsife College of Letters, Arts and Sciences, University of Southern California, Los Angeles, CA, United States; 4 Department of Sociology, Colorado State University, Fort Collins, CO, United States; 5 Institute for Research in the Social Sciences, Colorado State University, Fort Collins, CO, United States

**Keywords:** ideation, knowledge integration, research collaboration, research question generation, team science, transdisciplinarity, workshop design

## Abstract

**Introduction:**

Addressing “wicked problems"—complex societal challenges without clear boundaries or solutions—requires deep integration of knowledge across disciplines and sectors (transdisciplinary collaboration). While existing frameworks and toolkits support transdisciplinary collaboration, most interventions focus on established teams rather than the ideation stage.

**Methods and materials:**

This study introduces the Question-Collaboration Workshop, designed to stimulate transdisciplinary thinking and foster new research teams through collaborative question generation. The workshop includes four activities: (i) the Knowledge Creation Framework to make the scientific processes explicit; (ii) Buzzword Networking to build trust and a shared language; (iii) Four Layers of Questions to iteratively combine research questions across disciplines; and (iv) the Known-Unknown Matrix to identify knowledge gaps and actionable next steps. Two workshops were implemented at U.S. land-grant universities with researchers from diverse fields. Pre- and post workshop surveys and facilitator observations were analyzed qualitatively.

**Results:**

Both workshops seemed to successfully generate novel transdisciplinary questions and initiate new collaborations. Participants reported greater awareness of disciplinary perspectives and enthusiasm for future engagement. Challenges included time constraints, scaling activities for larger groups, and clarifying the Known-Unknown Matrix. Feedback emphasized the need for extended sessions, broader disciplinary representation, and follow-up activities to operationalize ideas.

**Discussion:**

Findings suggest that research questions can serve as a powerful catalyst for transdisciplinary collaboration. The workshop supports team science competencies such as trust-building, shared language, and co-creation of ideas. Limitations include small sample size and lack of longitudinal follow-up. Future work should refine activities, assess scalability, and evaluate long-term impacts on team formation and knowledge production.

**Conclusion:**

The Question-Collaboration Workshop demonstrates promise as an intervention to foster transdisciplinary thinking at the ideation stage. By leveraging researchers' intrinsic curiosity, this approach can help assemble diverse teams to address complex societal challenges.

## 1 Introduction

Transdisciplinarity or deep knowledge integration of disciplines and/or sectors has been lauded as one strategy to help solve “wicked problems” (Hou et al., 2022; Worosz, 2022; Yeung et al., 2021; Rittel and Webber, 1973). Wicked problems are deeply complicated challenges that lack clear boundaries or definitions, and cannot be solved with a single, straightforward, or ideal solution (Rittel and Webber, 1973). For example, Corona Virus (Schiefloe, 2021), Diabetes (Kerr and Glantz, 2020), and social-ecological systems (Caldas et al., 2019). These problems require bringing a diverse set of people together (teams) to tackle them from many angles and perspectives. The science of team science (SciTS) investigates the scientific principles, guiding specifically the teaming aspect within such complex research teams (Hall et al., 2018; National Academies of Sciences, Engineering; National Research Council, 2015). Thus a SciTS question arises: How do we help teams think transdisciplinarily to truly ideate across disciplines to solve these wicked problems? Please see Table 1 for a glossary of terms. To begin to answer this question, we created a Question-Collaboration Workshop to help researchers ideate across disciplines or think transdisciplinarily, to assemble new research teams around wicked problems. The goal of the workshop was to help researchers build their cross-disciplinary skills by developing research questions across disciplines. To do this, we intentionally brought together researchers from widely different disciplines, encouraging combinations that went beyond any shared knowledge domain. We present the workshop including new frameworks and tools, along with two case studies that highlight the power of research questions to help create new collaborations.

**Table 1:** Glossary of terms.

Term	Definition
Wicked problems	Deeply complicated challenges that lack clear boundaries or definitions, and cannot be solved with a single, straightforward, or ideal solution (Rittel and Webber, 1973)
The science of team science (SciTS)	A field that investigates the scientific principles, guiding specifically the teaming aspect within such complex research teams (Hall et al., 2018)
Thinking transdisciplinarily	Ideating across disciplines
Cross-disciplinary	Spectrum of collaborations that include more than one discipline (multi-, inter-, trans-disciplinary) (Hall et al., 2012)
Multidisciplinary	The sequential or additive combination of ideas or methods from two or more disciplines or fields to address a problem (Hall et al., 2012)
Interdisciplinary	The integration of perspectives, concepts, theories, and methods from two or more disciplines (Hall et al., 2012)
Transdisciplinary	“Research entails not only the integration of discipline-specific approaches, but also the extension of these approaches to generate fundamentally *new* conceptual frameworks, hypotheses, theories, models, and methodological applications that *transcend* their disciplinary origins, with the aim of accelerating innovation and advances in scientific knowledge” (Hall et al., 2012)
Cross-sector collaborations	Working with partners external to academia such as government, industry, community partners, patients, etc. (Faupel-Badger et al., 2022; National Academies of Sciences, Engineering)

Ideating across disciplines or knowledge integration takes three main forms: multidisciplinary, interdisciplinary, and transdisciplinary (Hall et al., 2012). The premise of cross-disciplinary research is “that each team member contributes unique knowledge, methodological approaches, conceptual frameworks, and theories, which collectively contribute to the advancement of scientific innovation and generation of new knowledge” (Hall et al., 2012). A multidisciplinary approach is the sequential or additive combination of ideas or methods from two or more disciplines or fields to address a problem. Interdisciplinary approaches involve the integration of perspectives, concepts, theories, and methods from two or more disciplines. Transdisciplinary “research entails not only the integration of discipline-specific approaches, but also the extension of these approaches to generate fundamentally *new* conceptual frameworks, hypotheses, theories, models, and methodological applications that *transcend* their disciplinary origins, with the aim of accelerating innovation and advances in scientific knowledge” (Hall et al., 2012). Transdisciplinarity is not only multi-discipline but also multi-sector: (Regeer and Bunders 2009) clarify “there is co-operation not only from the traditional knowledge institutes such as universities, but also from citizens, employees of businesses, the government, etc.” “Real world, tangible problems” or wicked problems are the starting point for transdisciplinary research (Regeer and Bunders, 2009).

This need for transdisciplinary thinking has led to the development of a variety of frameworks and strategies to support teams in embarking on a transdisciplinary effort. While researchers are highly trained in the theories, research methodologies, and frameworks within disciplines, few researchers have any formal training in integration across disciplines and methodologies (Mather et al., 2023). Aiming to fill this gap, scientists from across the disciplines have explored and compiled a variety of resources to support transdisciplinary teams (e.g., Worosz, 2022; Fam et al., 2018; Lang et al., 2012; Mather et al., 2023). These resources include frameworks for approaching different types of transdisciplinary problems [(Bammer 2020) collated nine in her blog], case studies of knowledge co-production around particular problems in unique contexts (Ahlstr'´om et al., 2020; Lang et al., 2012; Witinok-Huber et al., 2025), and broadly relevant tools that work across different types of problems. For example, the Toolbox Dialogue Initiative uses structured dialogue to impact mutual understanding, team communication, and decision-making (Hubbs et al., 2020), and td-net toolbox provides a variety of methods and tools that can be adapted in a variety of knowledge co-production efforts (Pohl and Wuelser, 2019). Most recently there have been efforts to expand the development and testing of team interventions and a variety of toolkits to help bring together the often fragmented work on inter- and trans-disciplinarity (ITD) (Laursen et al., 2024; Rolland et al., 2021). Many efforts have primarily aimed to assist existing research teams in synthesizing knowledge and advancing integration, or in codifying practices that already facilitate such outcomes. For example, Route to Identifying, learning, and practicing interdisciplinary and transdisciplinary team Skills to address difficult Environmental problems (RISE) is a structured process that “helps professionals with different expertise learn from each other by repeatedly asking team-developed questions” (Mather et al., 2023). The first step in the RISE process is “assemble team,” but it does not provide details on how to do that (see Figure 3 in Mather et al., 2023). In general, less attention has focused on developing tools and workshops in the nascent ideation stage, designed specifically to help researchers think transdisciplinarily no matter the topic area and to assist scientists to collaboratively explore the question formation phase of the scientific process (Newman, 2024). (Newman 2024), recently in a systematic review, raised an important question that needs more attention: “how to motivate researchers to conduct interdisciplinary research in the first place?”

To fulfill this need, we created the Question-Collaboration Workshop that invites researchers from any discipline to share and combine their research questions in an iterative cycle with researchers in other disciplines. We hypothesized that interacting with and combining research questions would help researchers think transdisciplinarily and assemble new teams through the process of question generation and refinement. In Materials and methods, we present the four activities in the workshop with information for use and customization (see Section Workshop activities). We also provide the workshop flow (including workshop surveys) for two different iterations of the workshop at two public land-grant universities (see Section Workshop flow at two universities). The Results discuss findings from the workshop surveys, including anonymous observations and reflections from workshop participants as case studies. Based on the two case studies, participants reported that they enjoyed the workshops and thought transdisciplinarily. We saw the formation of new transdisciplinary teams. They also requested more time and more disciplines for future workshops and provided feedback on the activities and workshop structure and flow. Future work can improve upon these activities, test our hypothesis at more institutions, and build upon this work to help new teams assemble around novel questions generated through cross-disciplinary interactions.

## 2 Materials and methods

The goal of the two workshops was to form new research teams merging data science and specialized fields (University A), and artificial intelligence (AI) and specialized fields (University B), supported by a data science institute and an office of research development, respectively. All participants were asked to bring their specific questions, problems, and methodologies, while also being reminded to be open to going beyond their common domain knowledge. At University A, the data science institute sent out an invitation through its listserv inviting researchers to think transdisciplinarily to build research teams that harness the multidisciplinary expertise across campus and cultivate the discipline of data discovery (the goal of the data science institute). Further, special invitations were sent to data science researchers to ensure there were some in the room. At University B, the office of research development launched a newer initiative of AI (artificial intelligence) + X For Good, with the X standing for the topics of health, education, and environment, to merge the topics with AI expertise (the goal of the initiative). University B recruited a cohort of interested researchers and the authors were contracted to bring this workshop to help form these new teams. Both workshops aimed to create questions that develop data science tools and answer questions and solve problems in other fields; merging data science and specialized topics. At University A, we gathered and qualitatively analyzed the novel questions formed in the workshop as part of workshop improvement. At University B, we deployed the workshop and did not capture their questions for analysis. For both workshops, the invitations spanned a wide-variety of disciplines and the workshop was intentionally field agnostic.

### 2.1 Workshop activities: new frameworks and tools to think transdisciplinarily

**Figure 1:**
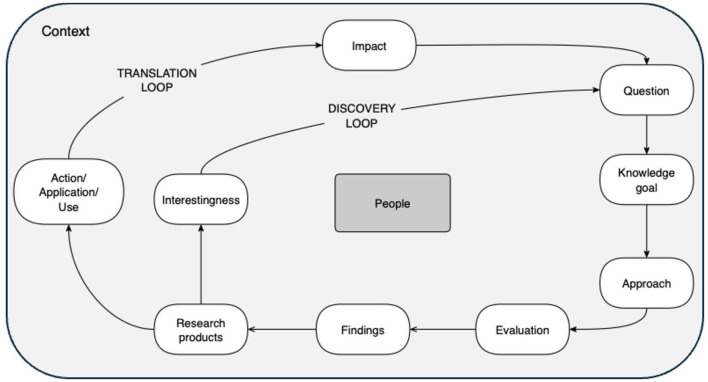
Knowledge Creation Framework (KCF) Version 1 (Boguslav et al., 2025).

The Question-Collaboration Workshop is organized around a new framework (Figure 1), the Knowledge Creation Framework, which elaborates 11 components of the cycle of scientific inquiry (Boguslav et al., 2025). Participants are guided through a series of activities designed to move researchers through iterative cycles of sharing knowledge, posing questions, and refining questions based on interaction across diverse expertise. These activities culminate in the dual creation of refined questions and potential new teams invested in answering those questions.

#### 2.1.1 Knowledge Creation Framework

The Knowledge Creation Framework (KCF) (Figure 1) is a boundary-spanning object created to help researchers from different disciplines make explicit their underlying scientific framework for their project (see Table 2 for an explanation of the components and Table 3 for details on how to use the KCF) (Boguslav et al., 2025). The KCF itself is also a tool to help researchers ideate and collaborate. The goal of the framework is to make explicit the steps of the scientific process, and thus aid teams in collective conversations about tacit assumptions and approaches, making explicit choices rather than assuming shared understanding (see “How does it work?” in Table 3). The ideal use of the KCF is as a living document: to articulate a new research project and refer back to as research progresses and changes (see Supplementary Figure S5 for a fillable table version of the framework). It can be used by one person or a team of people. In team science, explicitly documenting each component can foster a shared understanding and ownership of the project. Further, it can help bridge thought-styles, disciplines, sectors, and epistemologies of science and research through interactive use to gain agreement across team members within each of the 11 components.

**Table 2:** The components of the Knowledge Creation Framework with definitions.

Component	Definition
Impact	The role of science and technology in society
Context	The complex social, organizational, political, and technological milieu; external factors
People	The human contributors to the research
Question	Overarching driving research curiosity
Knowledge goal	Actionable next step from the question that drives the research
Approach	The systematic strategy or framework employed to investigate a research problem, including the underlying rationale, design, and methodological choices
Evaluation	The systematic assessment of a program or activity's processes and outcomes to determine its effectiveness and inform future improvements
Findings	The results or insights derived from a study
Research products	All things produced
Interestingness	The expected or unexpected that piques curiosity and leads you to new questions
Action/ Application/ Use	How the research products impact society through action, application, and/or use

**Table 3:** Knowledge Creation Framework information for use.

Guiding question	Information
What is Knowledge Creation Framework?	A boundary-spanning object created to help researchers from different disciplines make explicit their underlying scientific framework for their project
When should the method be applied?	At the beginning of new projects to help with project planning and design. This can also be revisited often to remind, revise, update, or continue along the cycle
What is made possible?	Explicit documentation of the scientific process for a problem or project. Allows everyone to ensure agreement on ideas and scope of project. Fosters shared understanding and ownership of the project. A required focus on the impact, context, evaluation, people, question, and application of the project
How does it work?	Introduce the goal of the Knowledge Creation Framework to help people document their scientific process for a project The following sequence is recommended; however, the iterative nature of the process allows for flexible ordering (1 and 2 are interchangeable): 1. Identify the **impact** or the why of the project in the world at large. Impact is at the top to highlight its importance. 2. Determine the **context** of the work including the people, the organizations, the funding, the disciplines involved, etc. 3. Identify the **people** needed including researchers and stakeholders. 4. Determine metrics for **evaluating** that the impacts are achieved or reached. 5. Determine **questions** of interest. 6. Identify **knowledge goals**, the next actionable steps, for the project (e.g., the need for an innovation in the field to overcome a barrier). 7. Determine how to **approach** the project to answer the questions. 8. Determine how to keep track of **findings** from the approach. 9. Determine what **research products** will be created from the findings and approach. 10. Identify what may be **interesting** about the results that could lead to new questions (**the discovery loop**). • What new questions do people have? • What impact will these have? • Connects back to 1–5 11. Identify how the results may be **acted upon by applied** and/or **useful** to the stakeholders and research community (**the translation loop**). Relate this to the impact of the project and context. • What is the impact of the results? • How do we apply and communicate them? • What role does context play? • What new impacts and contexts can the project have? • What new questions do we have? • Connects back to 1–5 See Supplementary Figure S5 for an example matrix to fill out for your research
How are thought styles bridged?	The Knowledge Creation Framework provides a forum and framework for bridging thought-styles, disciplines, sectors, and epistemologies
What's the output/outcome?	An explicit project plan that can be revisited often to remind, revise, update, or continue along the cycle. It helps develop a comprehensive shared understanding of and ownership over a project
Who participates in what role?	All members of the project team participate ideally including project management, collaborators, and stakeholders. A facilitator can moderate the discussion
What do I need to prepare?	**Space:** Table and chairs for writing and collaborating **Materials:** Copy of the Knowledge Creation Framework, paper, pens, computers if digital **Tips and traps:** • The order does not necessarily matter, although we stress the importance of the impact, context, people, evaluation, question, and the action/application/use to the world at large • Beginning with impact, context, people, evaluation, and question will foster the rest • Revisiting the framework created will be helpful for the project in the long run. This should be a living document **Riffs and variations:** • There are many activities for each step of the framework and for the connections between each step of the framework

For formal instructions for how to use the framework please see Table 3. Please note that the framework itself is a work in progress (version 1) (Boguslav et al., 2025). It was created after the first iteration of the workshop at University A and thus, it was only used in the workshop at University B.

#### 2.1.2 Buzzword Networking

Buzzword Networking is an informal activity designed to foster interdisciplinary connections by encouraging participants to guess one another's key terms or “buzzwords” (see Table 4). It falls under the People component on the KCF. At the outset of a project or workshop, it is essential to facilitate introductions to foster team affect—defined as “the development of empathy, affiliation, and rapport between members on the basis of shared regard for the other members of the translational team (TT)” (Brasier et al., 2023; Lotrecchiano et al., 2020). This is especially important for team-based communication and building trust, and should be developed early in a project team (Mendell et al., 2024). The activity involves participants anonymously recording their selected buzzwords, redistributing them among the group, and subsequently engaging in discussions to infer the origin of each buzzword without making direct inquiries. The goal is to help participants wake up through movement, think critically through lively discussions, and think transdisciplinary through the process of creating a shared language (Hall et al., 2018; Hilton and Cooke, 2015; Brasier et al., 2023; Mendell et al., 2024).

**Table 4:** Buzzword Networking information for use.

Guiding question	Information
What is Buzzword Networking?	An informal activity designed to foster interdisciplinary connections by encouraging participants to guess one another's key terms or “buzzwords”
When should the method be applied?	At the start of new group formation or the beginning of a workshop to help people get to know each other informally to foster trust and communication
What is made possible?	Open, generative conversations unfold while everyone gets to know each other informally. Group trust and a common language begin to form, breaking down the potential communication barrier in multi-disciplinary settings. This activity fosters learning how other fields and people relate to each person's buzzwords. Movement also helps participants wake up. Lively discussions help participants begin thinking critically. It begins the process of thinking transdisciplinarily
How does it work?	1. Introduce the goal of the activity: to help people get to know each other to build trust and communication 2. Each participant writes down 3–5 buzzwords on index cards. (5 min) 3. Facilitator collects all index cards, shuffles them, and returns 3–5 random index cards to each participant (2 min) 4. Participants read over the cards and walk around to learn about each other to match buzzwords to people. Informal groups may form (20–30 min—depends on group size) • If participants think they have a match, they can write the name on the back of the index card • Participants cannot ask others what their buzzwords are or ask for departments • Participants should not share if someone was correct 5. **Optional:** in the whole group, each participant introduces themselves and their buzzwords to confirm answers (10–20 min—depends on group size) 6. **Optional:** reflect on the exercise (5–10 min) • How did you match people to buzzwords? • Do you feel like you got to know everyone? How? • Based on this, how do you feel about working with people in the room compared to the start? • Thoughts on language used in conversations compared to buzzwords
How are thought styles bridged?	Everyone is engaged in getting to know each other by solving a problem or challenge (matching buzzwords to people) however they see fit
What's the output/outcome?	Participants will be warmed up and ready to think critically and think beyond their own discipline
Who participates in what role?	All group members can participate, providing buzzwords, and matching them. A facilitator is useful to return random index cards and keep time, especially for large groups
What do I need to prepare?	**Space:** chairs and tables for participants to write down their buzzwords; space for participants to wonder around to meet people face-to-face **Materials:** index cards, a set of identical pens for everyone to remain anonymous **Tips and traps:** • Encourage participants to rotate to meet as many people as possible • Ensure that everyone is meeting the whole group • Shuffle the index cards well and hand out randomly • Ask participants to speak up if they get their own buzzwords in the random hand out. Switch their cards as soon as you can **Riffs and variations:**• Use another fact about participants instead of buzzwords (e.g., answers to the question “what brought you here?,” “what is your passion?,” etc) • Assign groups ahead of time for participants to talk to instead of informal groups (for large groups especially). Facilitator can rotate groups as they see fit

Research itself is solving a puzzle and communicating it (Kuhn and Hacking, 2012). Creating a puzzle to solve—matching buzzwords to people—caters to the research process and facilitates communication and critical thinking. Participants inquire and converse with peers to become familiar with the research language used by each individual. Buzzwords, themselves, provide insights into the main topics of interest for researchers; a memorable snapshot of their work. Further, the activity is informal intentionally to help foster natural connections and conversations. By the end of the exercise, a collective terminology and deeper comprehension among participants start to emerge.

There is lots of flexibility with this activity, such as changing the buzzwords to answering a question or assigning groups ahead of time. The activity is designed to facilitate interaction with multiple individuals without requiring comprehensive engagement with every participant, which is impractical in large groups. No matter the form of the activity, People are at the center of the KCF because it shapes the research. Investing time for participants to build interpersonal connections, foster team cohesion, and develop a shared vocabulary has been shown to enhance long-term productivity (Bennett et al., 2018; Brasier et al., 2023; Mendell et al., 2024). Buzzword Networking offers a practical means to achieve this.

#### 2.1.3 Four Layers of Questions

Four Layers of Questions promotes cross-disciplinary thinking and collaboration by prompting participants to explore questions and perspectives outside their primary research fields (see Table 5 for the detailed activity). The focus for this activity is on the nascent ideation stage, combining the People and Question components of the KCF. The activity involves an iterative cycle of researchers continuously combining their research questions to generate and refine new transdisciplinary questions: starting with single research questions, to new double questions from two researchers, to quad questions of two double questions, to transdisciplinary larger group questions. The task begins simply with researchers writing down their current research questions, which usually are top of mind in general (Firestein, 2012; Kuhn and Hacking, 2012; O'leary, 2004; Boguslav et al., 2021). These questions both capture the current work of researchers succinctly, but also excite them. This good energy and passion tends to have positive affects on people in the activities (Mageau and Vallerand, 2007). With this momentum, all questions are shared with the group to help form pairs for the next cycle of double questions: participants “combining” their single questions into one new question. This cycle continues for the quad questions and so on. The intended outcome is the generation of novel transdisciplinary questions–and consequently new collaborations–that integrate diverse perspectives in innovative and meaningful ways.

**Table 5:** Four Layers of Questions information for use.

Guiding question	Information
What is Four Layers of Questions?	An activity that prompts participants to explore questions and perspectives outside their primary research fields. The activity involves an iterative cycle of researchers continuously combining their research questions to generate and refine new transdisciplinary questions
When should the method be applied?	At the start of group formation around a specific topic to find interested experts and questions
What is made possible?	The formation of transdisciplinary groups around specific topics with specific questions of interest to all group members. Forming transdisciplinary groups is difficult due to differences in every discipline. This activity provides an inviting framework, the question, to help bridge these divides by experiencing different levels of interaction with other disciplines
How does it work?	Introduce the goal of the activity: to harness multidisciplinary expertise across campus to build research teams to study a topic(s). Also to help people think and experience transdisciplinary thinking. Choose a topic(s) of interest. (Time will depend on group size) 1. Multi-disciplinary thinking on a topic(s) (25+ min) • Everyone writes down a question from their field and expertise to the topic(s) on a sticky note with names on them (5 min) • Share all questions with the group (5+ min) • Stick all the questions to a wall or flipchart paper so people can walk around and see them (5+ min) • Everyone stands near a question (aside from their own) that interests them (5+ min) • Facilitator helps pair people based on questions of interest (5+ min) 2. Inter-disciplinary thinking on a topic(s) (20+ min) • In pairs, combine questions into one. (10 min) Feel free to produce a new question together that combines expertise. Write new questions on a sticky note with both names. • Share paired questions and paste to wall or flipchart paper for all to see (5 min) • Facilitator helps pair up groups to form quads (four people) (5 min) • Continue iterating on combinations until final groups are formed 3. Transdisciplinary Thinking on a topic(s) (25+ min) • Combine all groups with the same topic to find a big question that encompasses all expertise. (20 min) Combine all combined questions. Write them down on sticky notes with all names on them. • Share all big questions (5 min) 4. **Optional:** reflection: • How did we choose who to work with to combine questions? • Were we surprised that we combined all together in the end anyways? • Were we surprised at our end question? • How did the language of the questions change? • How did our language of talking with our groups change? 5. **Optional:** provide time for groups to continue working on big questions. Consider using the Knowledge Creation Framework and associated activities
How are thought styles bridged?	Everyone gets to bring their expertise, specifically questions, to the table while learning from and working with others with different expertise
What's the output/outcome?	New groups formed around a topic with questions of interest to all ready to go
Who participates in what role?	All group members can participate, providing questions, and combining them. A facilitator is necessary for the activity, specifically to help pair people and to keep time
What do I need to prepare?	**Space:** moveable chairs and tables for participants to work in groups; a wall to post sticky notes or big sticky notes; space for participants to see the questions **Materials:** sticky notes varieties (4 × 6 regular, 4 × 4 lined), medium felt tip pens/markers, ballpoint pens **Tips and traps:** • Facilitators are recommended to read about disciplinary thinking from (Morton et al. 2015) (Figure 1 especially) • Facilitator needs to ensure everyone walks around and sees all questions • Facilitator needs to keep time **Riffs and variations:** • The introduction to this activity can vary depending on the group. The facilitator can explain the full activity ahead of time or can leave it open for surprise, but needs to explain the goal of the workshop • The number of combinations of pairs of people or groups is up to the facilitator based on group size and time • The focus on questions could change to be strengths or anything else that can be difficult to combine

Four Layers of Questions automatically fosters collaborations through the process. The task is to synthesize all questions into one, with the goal that every participant contributes and holds a stake in the resulting question. The process of the activity showcases transdisciplinary thinking: generating *new* ideas beyond any one discipline; learning from and educating each discipline (Hall et al., 2012). Through each layer of questions (1, 2, 4, etc.), researchers experience multi-disciplinary, inter-disciplinary, and transdisciplinary thinking (Hall et al., 2012) as the groups grow larger. With larger groups, it becomes more difficult to simply combine questions, and so the group needs to integrate, reconstruct, and re-imagine the questions. These discussions can begin to foster team communication (e.g., shared common language) and a shared vision (Hall et al., 2018; Hilton and Cooke, 2015; Brasier et al., 2023; Mendell et al., 2024) for a new collaboration around the final combined question.

The goal of this activity is to spark collaborations by discovering what unites participants, whether shared questions, complementary skills, or aligned values. Four layers of strengths or values could help find groups of people with similar or different strengths or values depending on the goal of the project. This activity uses questions (or any other combining force) to create a space for creativity and collaboration to begin to form team communication and a shared vision.

#### 2.1.4 Known-Unknown Matrix

The Known-Unknown Matrix is a framework to explicitly think through what is known and what is unknown about a project or problem (see Table 6). Explicitly documenting what are (known) knowns and (known) unknowns can help ensure agreement on ideas and next steps or knowledge goals, respectively. Two previous transdisciplinary research frameworks discussed persistent uncertainty (Mitchell et al., 2018) and managing unknowns (Bammer, 2019), recognizing the need to think about the unknowns. This matrix can help provide starting directions for a project or knowledge goals.

**Table 6:** Known-Unknown Matrix Information for Use.

Guiding question	Information
What is Known-Unknown Matrix?	A framework to explicitly think through what is known and what is unknown about a project or problem
When should the method be applied?	It can be useful at any stage of a project. It can be used in the beginning of the project and then revisited, revised, and updated during the project
What is made possible?	Explicit documentation of what is known and what is unknown. Ensures agreement on ideas (known) and what else needs to be done (unknowns). Both can help provide a starting direction for a project. By thinking through the unknowns especially, next steps can become obvious
How does it work?	1. Introduce the goal of the Known-Unknown Matrix to document what is known and unknown about a project. See two example matrices in Supplementary Figures S3, S4 2. Insert the topic in the first row 3. Begin with what is known in terms of different categories such as question, impact, and people. (This can be combined with Four Layers of Questions to start with question at the top) 4. Fill in what is unknown about each of the categories 5. Discuss next steps based on this matrix • What do we need to know next? • How will we find the information? • Who will find the information?
How are thought styles bridged?	The Known-Unknown Matrix requires everyone to acknowledge what they know and what they do not, and how that fits into the larger project. Everyone brings their own expertise and thought styles
What's the output/outcome?	A clear shared understanding of what is known and unknown. Potential action items to explore the unknowns
Who participates in what role?	It is helpful to have a facilitator to support the process. Everyone can contribute to the knowns and unknowns
What do I need to prepare?	**Space:** tables and chairs for writing and collaborating **Materials:** copy of the Known-Unknown Matrix, paper, pens, computers if digital **Tips and traps:** • The first category for the left column should be one that is known and obvious to people to make the diagram intuitive **Riffs and variations:** • The left column can be replaced with other KCF components

The Known-Unknown Matrix can provide a summary of the project. It can be used on its own or in conjunction with the other activities. It can overlay the KCF asking what is known or unknown about each component (see two example matrices in Supplementary Figures S3, S4). It can also be used individually or in a group setting. Either way, ideally, it functions as a living document that is continuously updated to reflect evolving knowns and unknowns.

The final activity for the Question-Collaboration Workshop was the Known-Unknown Matrix to leave newly formed teams with knowledge goals or actionable next steps. At the conclusion of the workshop, the objective was to establish new transdisciplinary teams equipped with collaboratively developed questions and shared knowledge goals to pursue.

### 2.2 Workshop flow at two universities

We used these frameworks and tools in two workshops at two universities with the explicit goal to assemble new teams by helping researchers think transdisciplinarily through questions and collaborations (Question-Collaboration Workshop). Authors Boguslav and Cross created and co-led the workshops. Two facilitators were intentional so that one could facilitate while the other took notes on the participants, the discussions, and the program overall for improvements. Both universities (A and B) are public land-grant research universities and the workshops were for data science institutes to help increase collaborations and usage of the center resources. The first workshop at University A was in August 2023 in the morning and the second workshop at University B was in November 2023 in the afternoon (see Supplementary Figures S1, S2 for detailed agendas, respectively). Surveys were administered at both workshops and were analyzed for common themes and quotes to better understand the participants' motivations, experiences, and suggestions for workshop improvements.

#### 2.2.1 University A: data science

The focus of the University A workshop was creating new collaborations for data science focusing on images/imaging/data representation as an example topic. Eight researchers participated from a broad range of disciplines including microbiology, electrical and computer engineering, sociology, math and statistics, geography, ecology, and computer science. The majority of the participants did not know each other before the workshop.

**Figure 2:**
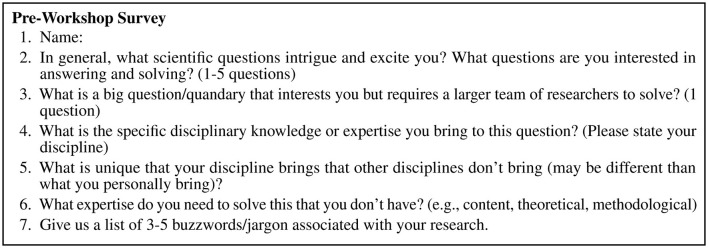
A pre-workshop survey sent digitally to participants ahead of time to begin thinking about questions and collaborations. A one page document. Only used at the University A workshop.

As part of registration we conducted a pre-workshop survey to prompt people to think about their questions and collaborations, determine a broad topic of interest to use as an example, and prepare for Buzzword Networking. The survey consisted of seven open-ended questions (see Figure 2). The last question provided the anonymous buzzwords for Buzzword Networking.

**Figure 3:**
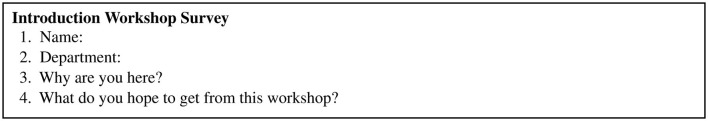
A brief introduction workshop survey. Half page document.

The workshop was 4.5 h in total (see Supplementary Figure S1 for a detailed agenda). During breakfast, everyone filled out a brief introduction workshop survey consisting of four open-ended questions (see Figure 3). The workshop content began with Buzzword Networking for 20 min to get to know each other. To facilitate broad interaction, participants were instructed to rotate among different individuals throughout the activity. Participants then spent 55 min working through the Four Layers of Questions activity, in which the details regarding the question-combination procedure were deliberately excluded. They had 5 min to write their single questions (eight questions); most people finished early. Everyone shared, and they were told they needed to combine their questions with one other participant. After 10 min, four new combined questions were created, with surprised/skeptical faces at the outset of this round. Then they were asked to combine again. Participants expressed surprise once again but demonstrated increased confidence in their ability to complete the task after having successfully done so previously. They went to work for 10 more minutes to produce two new combined quad questions. The final 15 min were devoted to combining all participants into a single group to develop a comprehensive question.

**Figure 4:**
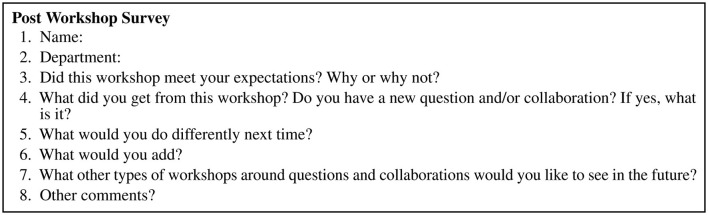
A brief post-workshop survey to assess learning and gather feedback. One page document.

After a 10 min break, the workshop turned to exploring collaborations focusing on the Known-Unknown Matrix. As a group, we filled out the matrix for the single comprehensive question with a focus on expertise or people (35 min). As a follow-on, each person individually filled out the matrix for their original single questions, shared them with a partner, and we discussed it overall as a group (45 min). The last 15 min were a final reflection on questions and collaborations. All participants filled out a one page post-workshop survey consisting of eight open-ended questions (see Figure 4). To provide time for open discussion and networking, the workshop ended with a networking lunch. This was a highly participatory workshop.

#### 2.2.2 University B: AI + X For Good

After a successful workshop at University A, we were asked to bring it to University B at one of their data analytics centers. In comparison to University A, we were only given 2 h total in the afternoon, the topic was previously chosen, the people already knew each other, and there were more people and more disciplines represented. (See Supplementary Figure S2 for a detailed agenda.) Note that authors Boguslav and Cross were external consultants for this workshop. The goal for the University B workshop was to form new collaborations around the topic of AI (artificial intelligence) + X For Good, with X standing for the topics of health, education, or environment, for future funding opportunities with the center. This workshop was the second in a series of workshops for a cohort for this center (approximately 15–20 people). A wide range of disciplines were represented including neurology, libraries, environmental health sciences, east Asian languages and literature, oral pathology (dentistry), public health, patient safety and quality, management, psychology, communication, etc. Note that we did not administer the pre-workshop survey.

The workshop flow at University B was similar to the first workshop, with major differences due to less time and a larger group. As people arrived and grabbed snacks, we asked them to fill out the brief introduction survey like at University A (see Figure 3). Since the researchers had met previously and for times' sake, we did a brief “getting to know each other” exercise (not Buzzword Networking), in which participants shared their name, department, and a personal passion in 15 min. We then jumped right into the Four Layers of Questions: write down a question and your X on a sticky note (5 min). In this iteration, the complete activity was explained prior to commencement, including the rationale for combining questions, to ensure participants understood the process and its purpose. With a larger group, participants posted their sticky-note questions on a wall, allowing others to circulate, review the questions, and position themselves near those they found most compelling. We paired people based on this to write their double questions—combination of two people (10 min). Building on the paired questions, participants were instructed to organize into groups based on their common categories (X) and collaboratively integrate all paired questions (25 min). Note that the groups were of varying sizes based on interest in the X (3–10 people per group).

Following a 5 min break, we introduced the KCF to contextualize this activity within the larger scientific method. The call for this workshop was focused on the impact “for good.” Using three components of this framework—Impact, Question, and People—we asked each team to fill out the Known-Unknown Matrix for their team question (45 min). We saved 10 min at the end for a final reflection and for participants to fill out the post-workshop survey (see Figure 4) like in the University A workshop.

## 3 Results: case studies from two university workshops

Both workshops resulted in new ideas and collaborations amongst researchers. The Four Layers of Questions activity most prominently demonstrated transdisciplinary thinking. We also achieved a high survey response rate by integrating the surveys into the workshop activities. All observations and reflections are from discussions and reflections during the workshop, the workshop surveys, and observations by facilitators and authors Boguslav and Cross based on notes taken during the workshops by one of them. All quotes are anonymized from the workshop surveys, unless otherwise noted.

### 3.1 University A: data science

Together the group of eight people created multiple multi-disciplinary and transdisciplinary questions around the common broad topic of images/imaging/data representation, based on reviewing all participants answers from the pre-workshop survey. Note that seven out of eight participants filled out all surveys (author and facilitator Cross participated in the activities but did not fill out the survey). Everyone seemed impressed and we reflected on the process focusing on surprises and language. Although the primary focus was on generating questions, new collaborations appeared to form effortlessly. By the end of the workshop, the participants all knew each other through all four activities and two new transdisciplinary teams formed with questions and actionable next steps (Knowledge Goals).

#### 3.1.1 Observations and reflections

During the Buzzword Networking, we saw researchers wake up through movement and think critically through discussions about buzzwords. We encouraged participants to rotate and meet additional individuals, as many were eager to continue conversations with those they had already engaged. Despite allocating 20 min for this activity, participants requested additional time, indicating high levels of engagement and enjoyment.

Researchers came up with questions very quickly. Almost no one needed the full 5 min to write their own single questions. Note that more time was needed for combining, and most completed the task within the allotted time (10–15 min). Even still, participants seemed surprised and potentially a little skeptical that they could combine their questions. As questions were combined in each round, we observed a decrease in skepticism along with an increase of confidence. Participants saw the pattern as we kept going and leaned into the activity. In the last two rounds, participants began collaborating right away to merge their questions. The exercise revealed that researchers not only engaged in transdisciplinary thinking but also adopted this approach with notable ease.

Some confusion arose regarding the use of the Known-Unknown Matrix, as participants were uncertain about the distinction between “known” and “unknown.” For example, several questioned whether writing something down implied that it was known. The overarching concept is to differentiate between what is already understood (known knowns) and what is missing or requires further investigation (known unknowns) (Boguslav et al., 2021, 2023). This clarification seemed to improve participants' comprehension of the activity. Future iterations need to include revisions to clarify these distinctions.

Overall, participants valued the workshop, their expectations were met, and they demonstrated team science competencies. The reflections and survey responses provided critical insights into participants' motivations for attending, the benefits they perceived, potential improvements, and their preferences for future offerings. The participants came to the workshop to find collaborators (with data), to meet people with common interests, to find out what interesting work others do, to learn about questions, to learn about the center's data and resources, and to learn how their resources could help others. They hoped to gain understanding of applications of their work, collaborators, connections across campus, insights on setting up a collaboration workshop, a better sense of the center, and strengthened relationships with others and the center. Overall, these expectations were met and more (all participant quotes from the Post-Workshop Survey at University A): “Interesting process to converge on a topic among a group with broad interests.” Some did not have expectations but thought “it was more engaging than they expected” and that “it was helpful to see others struggling with the same issues.” Not all participants left with new collaborations and/or new questions, but they learned what others do, how they think, and how we all can overlap. Participants realized that questions they understand apply to new problems. They thought about and practiced how to communicate across disciplines. All of these are goals and competencies of team science (Brasier et al., 2023; Lotrecchiano et al., 2020; Mendell et al., 2024). In general, participants expanded several of their existing questions and collaborative ideas, considering new directions inspired by workshop interactions and discussions.

Overall, we received positive feedback on the workshop, along with concrete suggestions for improvements and future workshops (all participant quotes from the Post-Workshop Survey). They requested more time for the various elements of the workshop (e.g., the Buzzword Networking in the beginning) and a broader representation of disciplines suggesting physics, chemistry, etc. Participants expressed interest in dedicating additional time or hosting follow-up workshops focused on translating new collaborations and questions into actionable research plans. They emphasized the challenge of operationalizing innovative ideas and sought guidance on leveraging available resources to address these questions (“operationalizing cool ideas is harder than coming up with them”). Participants suggested tailoring future workshops to specific stages of the research process (e.g., initiation through conclusion) and career levels (e.g., graduate students to faculty). They also recommended adopting a hackathon-style format (Falk et al., 2024) to allow more time for developing solutions to identified questions. We plan to explore these ideas in future work.

### 3.2 University B: AI + X For Food

The group at University B was very diverse and large, which came with its own opportunities and challenges. Sixteen introduction surveys and thirteen post-workshop surveys were collected.

#### 3.2.1 Observations and reflections

Participants attended the workshop to “network + learn + collaborate” (all quotes in this section are participant quotes from the Post-Workshop Survey at University B). They wanted to learn about AI, translational research and science, the center itself, and “how to proceed with an existing interdisciplinary group.” They hoped to gain a better understanding of AI, new ideas to leverage AI, new ideas for teaching and research, “how to knowledge,” “be able to make the right questions for my research,” ideas about next steps for collaboration and ideas, get to know collaborators, and “how to start a project fundable.” Overall, the workshop met most people's expectations: giving people ideas, a network, and funding opportunities.

It was important to begin with getting to know each other to build team (the cohort) affect (Brasier et al., 2023; Mendell et al., 2024). Even though most people already knew each other, the activity served as a reminder of names, as a transition into a new space and new activity, informed everyone of the disciplines in the room, and continued to build trust amongst the group. To promote transparency and trust, external consultants and authors Cross and Boguslav explained the Four Layers of Questions exercise to participants before initiating the activity. While this preparation reduced participants' initial surprise, full comprehension seemed to only emerge through direct engagement. These observations underscore the importance of understanding the audience and establishing trust before initiating such activities.

The University B group was significantly larger (at least double) than the University A group creating challenges with facilitation. Initially, the plan was for all participants to share their single question, as implemented at University A; however, the large number of participants and the presence of three discussion topics rendered this approach impractical. Instead we had them paste their questions on the wall by each topic and walk around to review them. The participants required reminders to visit each note because many of them began conversing with one another rather than engaging with the posted questions. Additionally, pairing participants proved more challenging than at University A, as the larger group size necessitated creating more than eight pairings. To streamline the pairing process, participants were asked to position themselves by the sticky note that captured their interest, enabling pair formation through topic alignment. To our surprise, some pairings were mutual, while others took a little more work. Overall though, everyone was paired to write their double questions. The final step to the larger groups was easy because everyone combined based on their X. Everything required more time than we allotted and more facilitation than we realized. Future work on how to scale this workshop and stick to time is necessary.

The addition of a draft KCF served to contextualize the activity and it also introduced unintended confusion among participants. Although the framework was presented without direct application, we proceeded to the Known-Unknown Matrix activity using three of its components. While most researchers are implicitly familiar with the principles underlying the KCF, they rarely encounter it articulated as an explicit framework akin to the scientific method. Positioning the KCF at the outset of the workshop could have served as a motivating structure for identifying questions and collaborators in alignment with the workshop's emphasis on generating impact “for good,” while also foreshadowing the subsequent use of the Known-Unknown Matrix. Similar to observations at University A, participants initially found the Known-Unknown Matrix challenging to interpret; however, with additional explanation, they were able to complete it. Despite these difficulties, most participants concluded the workshop with actionable knowledge goals related to ideas, collaborations, and, in some cases, funding opportunities from the center.

The workshop surfaced actionable insights for the center to continue to meet the needs of the participants. There were many diverse disciplines focused on the three X's represented in the room and almost no AI experts. Some participants wanted more diversity—“not enough participants” for divergence of ideas—and some wanted less—this workshop “provided insights into the divergence phase, [but] did not (understandably) allow for the convergence [of ideas] that we naturally want.” However, many participants were not sure how feasible their questions or ideas were without AI researchers. They suggested seminars about AI applications, methods, limitations, and policies or mixers with AI experts. Using this information, the center identified this as the theme for their next cohort meeting.

Some participants requested more time for collaborations and questions with ideas for other workshops in the future. For example, “longer workshop which leads to a white paper for a grant.” Digital capture was suggested to help those with other time commitments such as teaching. They also requested more focus on research strategy. Overall participants thought and learned more broadly from other disciplines through this workshop: “I saw a use case beyond my own vision of a project”; thinking transdisciplinarily. Not only did the participants gain valuable insights into questions and collaborations, but also the center learned more about the needs and wants of their research population more generally.

### 3.3 Tracking transdisciplinarity: analyzing the novel questions from University A

At University A, we had access to all questions and we analyzed the evolution of the questions through the different layers of the workshop. The questions zoomed into greater levels of specificity within disciplines from the single to double to quad questions, but then zoomed out in the large group questions. Note that we will not provide the participant questions to protect them and their research. Because the workshop aimed to bridge data science methods with other disciplines, all participants contributed a methodological question drawn from challenges they were facing within their respective fields (single questions). All of the questions were about identifying key variables and relationships to drive understanding and/or change. Most questions began with “how” and prompted the need to find or create methods to answer them. The double questions (paired question formation) became more specific to a field and application topic. More specific variables, relationships, and outcomes were stated in the format: “how can we use [methodology] to answer [topic question]?” with the methodology from one field and the topic question from another. The Quad questions (pair of pairs, four people) continued to zoom in to combine four fields: “how can we use [methodologies] to answer [topic questions] for [outcomes]?” These questions now included and combined multiple methodologies, topics, and outcomes. Interestingly, combining the full group into large questions led to zoomed out questions. For example: (1) “What variables have the most explanation/predictive value to inform future research? How do we figure that out?” and (2) “How do you determine the complexity of a data set?” (University A participants). It seems researchers are grappling with similar questions underneath their own and all have concrete research interests in them, no matter the field.

## 4 Discussion

We offer the Question-Collaboration Workshop as one answer to Newman's question of how to stimulate collaboration across research disciplines without the need for shared knowledge or field (Newman, 2024). Wildly divergent scientists who have never worked together before were able to come up with real problems and look for real solutions and opportunities to study together that touched on their specific fields. At University A, two new teams were formed, and at University B, the office of research development determined their next cohort meeting topic based on this workshop. The Question-Collaboration Workshop focuses on the People and Question components of the KCF and scientific inquiry. We hope to continue these efforts to help researchers shape their scientific inquiry and collaborations for real world impact.

Researchers are driven by research questions (Firestein, 2012; Kuhn and Hacking, 2012; Han et al., 2011; Pearl and Mackenzie, 2018; Walker, 1990; Smithson, 2012; Proctor and Schiebinger, 2008; Rubin, 2006; Martin and White, 2003; Bromberger, 1992; Regan et al., 2002; O'leary, 2004) and many research problems currently require collaboration across disciplines (Hall et al., 2018; Brasier et al., 2023; Wu et al., 2019; Wuchty et al., 2007). Combining these two ideas, we presented the Question-Collaboration Workshop that uses each person's driving questions to form new collaborations and think transdisciplinarily from the beginning and intentionally field agnostic. These activities support researchers who have not previously collaborated in cultivating a common language, building interpersonal trust, and explicitly co-creating novel ideas (established team science competencies Brasier et al., 2023; Lotrecchiano et al., 2020; Mendell et al., 2024). Further, these activities, especially the Four Layers of Questions, supported researchers zooming in and out of research topics (thinking transdisciplinarily). This aligns directly with social innovation theory in systems thinking (Zankl and Grimes, 2025). “Social innovation involves the novel reconfiguration of resources and ideas to address significant social challenges” (Zankl and Grimes, 2025). The Question-Collaboration Workshop activities help researchers create novel reconfigurations of topics and fields to drive new ideas to solve societal challenges.

To allow for these workshops to be repeated and replicated, we provided detailed instructions and variations for the workshop activities (see Tables 3–6) and two case studies with positive outcomes as examples (see the Results section). We encourage other institutions to use and adapt these in their own settings to form new transdisciplinary questions and collaborations for their topics of interest. Future work should include train the trainer modules to support facilitators at other institutions. Key design elements to consider for train-the-trainer modules include: (1) topic selection in conjunction with consideration of the invitation list and strengths across fields at the institution, (2) time allotment for activities related to number of participants, and (3) sequencing of additional workshops to build teams emerging out of the Question-Collaboration Workshop. Building trust, communicating across fields, and translating knowledge requires time and cannot be rushed. Feedback from participants suggested that the activities could use more time for discussion and that newly catalyzed teams expressed the desire for additional structured workshops to take the next steps in elaborating their questions, considering approach, and identifying potential funding opportunities. Note that this is just the first step in question building across fields. One such tool that can help the newly formed team “row in the same direction” is MIX: a tool to help individual disciplinary experts meld into an integrated interdisciplinary team (Mather et al., 2024). We also plan to continue building on these efforts to help newly formed transdisciplinary research teams integrate and succeed in their research efforts (e.g., iterating on the KCF).

The boundary conditions of the workshops are worth noting before organizing one of your own. In both universities, a topic that would draw researchers from across disciplines was selected before the workshop was advertised. Then, invitations were sent out to researchers from a variety of disciplines in order to seed the workshops with disciplinary breadth. For University A, a pre-workshop survey was used to determine a topic (see Figure 2). For University B, the center decided the topic. In both cases, we collaborated with the workshop host to define a topic that everyone could discuss or touch on no matter the discipline; i.e. transdisciplinary in nature. Future work could test if this workshop would work if no topic was decided ahead of time. We did not test whether fruitful collaborations might emerge without a clearly defined topic to attract a variety of researchers. Open questions include: would researchers come to such a workshop without a theme? Are they more likely to come if they knew what disciplines would be represented? Is there a minimum number of disciplines or participants needed for a successful workshop? Do certain disciplines need to be targeted specifically depending on the goal of the workshop or topics of interest? Participants had mixed feelings on the representation of disciplines in the room. Some felt like it was enough and some wanted more. For University B, AI researchers were identified as missing, but desirable partners.

Another unexplored area is time. How much time do these workshops require? Does the time of day matter? University A was in the morning for 4.5 h and University B was in the afternoon for 2 h. Further, many participants requested more time for different activities, including getting to know each other and the Four Layers of Questions. How do institutions balance the need for time with the demands on researchers for other things? Also, the more people at the workshop, the more time that is needed to integrate and share after each round of question posing. Future work could explore the relationship between time, activities, number of people, and goals.

Some participants requested more activities in general, especially ones that further the research efforts of the newly formed teams (e.g., continuing to other components of the KCF). What other activities could or need to be paired with this workshop? Hackathons (Falk et al., 2024), better understanding of a topic (like AI for University B), and funding opportunities were suggested by participants. These workshops themselves offered insights to the needs of the research participants. Also, the activities in the workshop can be updated. For example, the Known-Unknown Matrix activity was confusing for both Universities, and it was unclear at University B how to best use the KCF. Future work can think about improving upon the current activities in the workshop and other activities to add.

The primary limitations of this work were the small sample size, the focus on one topic area of data science, and the absence of follow-up with participants to determine whether the newly formed transdisciplinary research teams persisted and generated new knowledge. Future research should focus on developing strategies to evaluate both the workshops and their impact on researchers and team formation. We hope to implement this workshop at other universities, settings, and topics to strengthen these conclusions and assess its scalability. Notably, scaling up the number of participants while reducing the duration—from University A to University B—introduced challenges that must be considered in future iterations. A comprehensive assessment of workshop impact would require longitudinal follow-up with newly formed teams, which was not feasible in the current study. However, observations during the closing session indicated that participants exchanged contact information to enable future collaboration, and we documented participant recommendations through informal conversations and post-workshop surveys to support ongoing team development. Overall, further research is needed to refine evaluation methods, generalizability, scalability, and assess the long-term effectiveness of these workshops and activities on different topics and fields.

## 5 Conclusion

In conclusion, the Question-Collaboration Workshop demonstrated the potential to foster transdisciplinary thinking and new team formation at two universities. Questions seem to motivate researchers to collaborate. Initial findings suggest that these activities can catalyze valuable connections among researchers, even with limitations such as small sample size and lack of longitudinal follow-up. Future work should focus on evaluating long-term impacts, refining assessment strategies, and exploring scalability of the Question-Collaboration Workshop across diverse institutional contexts.

## Data Availability

The datasets presented in this article are not readily available because the original program evaluation did not contain consent to gather and share the data for research purposes. Requests to access the datasets should be directed to Mayla Boguslav, Mayla.Boguslav@med.usc.edu.
