# Supplementary material for: Thinking transdisciplinarily: Question-Collaboration Workshop to ideate across disciplines
Source: Front Psychol. 2026 May 5;17:1773669. doi: 10.3389/fpsyg.2026.1773669 (PMC13183573; doi:10.3389/fpsyg.2026.1773669)
Supplement: Supplementary file 1 [file Data_Sheet_1.pdf]

Supplementary Material

Please find the detailed agendas for each workshop and fillable forms that were used as handouts at the workshops.

1 SUPPLEMENTARY FIGURES

1.1 Detailed Agendas for Workshops

Workshop Version 1: Agenda (all interactive)

8:30 - 9:00 AM Breakfast Pastries

9:00 – 9:20 AM Get to know each other

9:20 – 10:15 AM Question exploration

10:15 – 10:25 AM Break

10:25 – 11:00 AM Collaboration exploration

11:00 – 11:45 AM Refining questions and collaborations

11:45 AM – 12:00 PM Final reflection

12:00 – 1:00 PM Lunch and Networking

Figure S1. Detailed Workshop Agenda Version 1. It was used as a handout at University A.

Workshop Version 2: Agenda (all interactive)

3:00 – 3:05 PM Introduction and Intro Workshop Survey

3:05 – 3:20 PM Get to know each other

3:20 – 4:00 PM Question exploration

4:00 – 4:05 PM Break

4:05 – 4:50 PM Refining questions and collaborations

4:50 – 5:00 PM Final reflection and Post Workshop Survey

Figure S2. Detailed Workshop Agenda Version 2. It was used as a handout at University B.

1.2 Fillable Forms

**Topic/ Problem/ Question**		
	Known	Unknown
Information		
Expertise		
People		

Figure S3. Known-Unknown Matrix Fillable Form Version 1. It was used as a handout at University A.

**Topic/ Problem/ Question**		
	Known	Unknown
Question		
Impact		
People		

INTERNAL

Figure S4. Known-Unknown Matrix overlaid with KCF Fillable Form Version 2. It was used as a handout at University B.

Knowledge Creation Framework
NAME: __________________

Category	Research
Impact	
Context	
People	
Question	
Knowledge Goal	
Approach	
Evaluation	
Findings	
Research Products	
Interestingness	
Action/Application/Use	

Created by: Mayla Boguslav, PhD and Jeni Cross, PhD

Figure S5. Knowledge Creation Framework Fillable Form. It was used as a handout at University B.
